# Perinatal healthcare inequities: A qualitative causal network analysis

**DOI:** 10.1002/pmf2.70021

**Published:** 2025-05-04

**Authors:** Monica A. Lutgendorf, Gayle Haischer‐Rollo, Teri R. Ryals, Torie C. Plowden, Erin M. Blevins, Carmen N. Spalding, Caitlin M. Drumm, Rasheda J. Vereen, Brelahn Wyatt‐Nash, Shara M. Fuller, Megan G. Musilli, Abigail Konopasky

**Affiliations:** ^1^ Department of Gynecologic Surgery and Obstetrics Uniformed Services University of the Health, Sciences Bethesda Maryland USA; ^2^ Department of Pediatrics Uniformed Services University of the Health Sciences Bethesda Maryland USA; ^3^ Department of Obstetrics and Gynecology Navy Medical Readiness and Training Command Bremerton Bremerton Washington USA; ^4^ Department of Pediatrics Naval Medical Center San Diego San Diego California USA; ^5^ Sharp Healthcare System San Diego California USA; ^6^ Department of Gynecologic Surgery and Obstetrics Naval Medical Center San Diego San Diego California USA; ^7^ Department of Medical Education Geisel School of Medicine at Dartmouth Hanover New Hampshire USA

**Keywords:** birth experiences, health inequities, qualitative

## Abstract

**Introduction:**

Persistent healthcare inequities exist within the Military Health System (MHS). The objective of this study was to use qualitative interviews to assess the intersection of operational and cultural experiences of military service with the lived experience of individuals on their use and experience of perinatal health care.

**Methods:**

This is an IRB‐approved qualitative study of the perinatal experience of servicemembers and TRICARE beneficiaries. A total of 36 semi‐structured interviews were conducted with individuals who delivered an infant within the last 5 years. Causation coding and deductive methods were used to generate a variable list of antecedent variables, mediating variables, and outcomes. Themes were organized into causal chains based on participant stories. A causal network was developed using cross‐case mapping to generate a thematic narrative from a systematic comparison of within‐case causal networks.

**Results:**

A complex detailed causal network was developed, depicting structural and social factors affecting birth experiences. Such causal relationships impact individual experiences to varying degrees. Antecedent variables included experiences of dismissal, lack of support, concerns about the pregnancy, knowledge of poor outcomes, family experiences, and systemic issues. Mediating variables included delays in care, lack of command support, fragmented care, microaggressions, and fear of medications and interventions. Outcomes included mistrust of the medical system, fear and anxiety, early cessation of human milk feeding, and decreased military retention.

**Conclusion:**

Comprehensive and logical pathways illustrate challenges faced by birthing individuals in the MHS. Results suggest that outcomes including mistrust, fear, anxiety, and early cessation of human milk feeding may be mediated by participant healthcare experiences and concerns.

## 1 INTRODUCTION

Healthcare inequities are preventable differences in the quality of healthcare and health outcomes in socially disadvantaged individuals, and these inequities can result in differences in healthcare access, disease occurrence, and healthcare outcomes [1]. Adverse perinatal outcomes affect birthing individuals and their children, with Black birthing individuals two to three times more likely to die from pregnancy complications compared to White birthing individuals [2]. Additionally, in the United States, children of Black, non‐Hispanic women have a disproportionately high neonatal mortality rate of 6.85/1000 live births which is a nearly threefold increased risk of neonatal death compared to the national rate of 3.69/1000 live births [3]. Persistent inequities also exist despite education, and the pregnancy‐related mortality ratio for Black birthing individuals with at least a college degree is five times as high as White birthing individuals with similar education [2]. Racial inequities in perinatal morbidity and mortality have been present in the United States for over 100 years and have links to structural and systemic racism impacting care [4].

The Military Health System (MHS) has an annual budget of $55 billion, and covers 89,000 births, with 75,000 births in the purchased care (civilian) market with Tricare insurance [5]. In the MHS, despite universal insurance coverage and stable employment, perinatal healthcare disparities and inequities exist, with Black pregnant individuals experiencing higher rates of cesarean delivery, admission to the intensive care unit, and maternal morbidity compared to White pregnant individuals [6]. Black, Asian Pacific Islander, and other races also experienced higher rates of cesarean delivery, postpartum hemorrhage, and severe maternal morbidity compared to White birthing individuals in the MHS [7]. Furthermore, causes of pregnancy‐associated deaths in active duty individuals varied by race, with higher rates of deaths from other pregnancy‐related causes, assault, and transportation accidents in Black birthing individuals [8]. Some studies have also identified healthcare inequities related to military rank (socioeconomic status), with higher military rank associated with shorter hospitalizations and lower in‐hospital mortality in patients with ischemic stroke admissions [9]. The neonatal mortality rate within the MHS is lower than in the United States overall and is comparable to other developed nations. However, neonatal racial inequities also persist in the MHS, placing infants born to Black service members at a significantly increased risk of death in the neonatal period even when accounting for socioeconomic status and universal healthcare [3].

Despite persistent perinatal healthcare inequities in the MHS, prior studies have not explored the lived experiences of birthing individuals and how experiences such as trauma, military deployments, and other stressors may affect birth experiences and outcomes. The purpose of this study was to conduct a causal network analysis to understand the antecedent and mediating factors that contribute to healthcare inequities.

## 2 MATERIALS AND METHODS

Participants included individuals who delivered newborns within the past 5 years at a military treatment facility or civilian facility using TRICARE insurance in the United States and at worldwide facilities (both military and civilian). Additional inclusion criteria included age of 18 years or older and English speaking. Participants were recruited using social media posts, email blasts, snowball sampling, and flyers posted at military hospitals. Individuals were recruited with maximum variation and homogeneous sampling to represent diverse viewpoints based on socioeconomic status and self‐identified race and ethnicity. Maximum variation was used to assess shared patterns between participants across a variety of perspectives, and homogeneous sampling was used to provide an in‐depth description of participant groups [10]. Participants reviewed a study information sheet prior to making an informed, voluntary decision to participate.

An interprofessional group of clinicians and a qualitative researcher convened to conduct this study using a grounded theory approach [11] to understand participant's birthing experiences. The study team included physicians, residents, a midwife, and a qualitative researcher. Reflexivity statements are included in Supporting Information S1.

Semi‐structured interviews were conducted from February 2023 to June 2023, and included questions related to participants’ birth experience, and prenatal and postpartum care. The data were part of a larger study focused on military beneficiaries’ birth experiences [12]. The interview guide was developed by the authors to investigate participants’ specific lived experiences during perinatal care and delivery to capture insights into potential healthcare inequities and is shown in Supporting Information S2. Interviews included open‐ended questions about their pregnancy experience, childbirth, postpartum care, newborn care, personal and family history, and military experiences as applicable. Though participants were required to have a birth within the past 5 years to be eligible to participate, they were not excluded from talking about earlier birth experiences that they deemed important. The semi‐structured interviews lasted between 45 and 90 min and were conducted with trained study investigators using Google Meet (Mountainview, CA). Participants gave verbal informed consent to participate in the study, including video recording and transcription using Google Meet software. All participants were free to end the interview early or decline to answer questions during the interview. At the end of the interview, participants were asked demographic questions including their age, self‐reported race and ethnicity, active‐duty service status, branch of service for themselves or their partner, rank for themselves or their partner, and travel time to their obstetric clinician and delivery location. Following the interview, participants were assessed for safety and provided counseling resources as needed. Participants were offered a $25 electronic gift card for their participation in the study. Interviews were conducted until thematic saturation was reached [13]. All interview transcripts were de‐identified.

Each interview was coded by a trained investigator to assess causal themes, with causation coding and deductive methods used to generate a variable list of antecedent variables, mediating variables and outcomes. These variables were then organized into a flow chart to determine what generally leads to what, and then further organized into the final causation model [14]. Causation coding allows researchers to understand participants’ rationale for why things are the way they are from their perspective [14]. Causation coding is based in attribution theory, which includes the causal explanations produced by participants related to their experiences and perspectives, and allows the generation of plausible causes of outcomes [14, 15]. Coding was completed as interviews were conducted, with two authors coding each interview. Following completion of the first four interviews, the team reviewed the coding and made modifications to the interview guide to ensure participant understanding and depth of responses. To ensure consistency and accuracy of coding, group discussions were held at approximately 4‐week intervals, and the interview guide was iteratively adjusted. Group discussions continued until consensus on coding was reached. Themes were organized into causal chains based on participant stories. A causal network was developed using cross‐case mapping to generate a thematic narrative from a systematic comparison of within‐case causal networks [14]. The steps of the causal network analysis are listed in Supporting Information S3. Initial coding and analysis was conducted, with attribution sequences plotted including antecedent conditions → mediating variables → outcomes. After initial categorization, similar outcomes were combined into major categories that led to specific outcomes, answering the ‘why’ question. The research team iteratively reviewed the causation coding to ensure accuracy, with cycles of codebook revision and iterative revisions of the causal network. Throughout the process the team reflected on their involvement as clinicians, military personnel, and mothers impacted their interpretation of participant narratives, with the team challenging these perspectives through discussion and finding opposing examples in the data. Interviews were conducted until saturation was reached with no new substantiative information obtained [10]. The study was approved by the Institutional Review Board of the Uniformed Services University of the Health Sciences and conforms with the U.S. Federal Policy for the Protection of Human Subjects.

## 3 RESULTS

A total of 77 individuals expressed initial interest in participation during the study period, with 36 individuals completing interviews, and 41 individuals declining participation or not following up to schedule an interview. Participant demographics are shown in Table 1. Most participants identified as non‐Hispanic White, active duty servicemembers in the U.S. Navy. Care was received in both military and civilian care settings with some individuals experiencing care overseas, and some individuals experiencing care in both military and civilian settings, either due to transfer from one system to the other or in different pregnancies.

**TABLE 1:** Background characteristics of military beneficiaries with deliveries in the past 5 years.

Characteristic	*n* (%) (*n* = 36)
Current age	
18–24 years	2 (5.6)
25–29 years	4 (11.1)
≥ 30 years	21 (58.3)
Self‐Reported Race and Ethnicity	
non‐Hispanic Black	7 (19.4)
non‐Hispanic White	17 (47.2)
Hispanic	4 (11.1)
Multiracial	5 (13.9.9)
Jewish	1 (2.8)
Asian (Indian)	1 (2.8)
Christian Middle Eastern	1 (2.8)
Indigenous	1 (2.8)
Active duty servicemember	34 (94.4)
Branch of Service (self)	
Air Force	8 (22.2)
Army	4 (11.1)
Navy	20 (55.6)
Rank/Rate (self)	
Junior Enlisted (rank E1–E5)	5 (13.9)
Senior Enlisted (rank E6–E9)	9 (25)
Junior Officer (rank O1–O3)	6 (16.7)
Senior Officer (rank O4–O6)	7 (19.4)
Family member of active duty servicemember	4 (11.1)
Branch of service partner (family members)	
Air Force	0
Army	1 (2.8)
Navy	2 (5.6)
Rank/Rate partner (family members)	
Junior Enlisted (rank E1–E5)	1 (2.8)
Senior Enlisted (rank E6–E9)	2 (5.6)
Junior Officer (rank O1–O3)	0
Senior Officer (rank O4–O6)	0
Time to primary OB clinician	
< 30 min or less	16 (44.4)
30–59 min	10 (27.8)
≥ 60 min	4 (11.1)
Time to delivery location	
< 30 min or less	19 (52.8)
30–59 min	8 (22.2)
≥ 60 min	2 (5.6)
Setting of Care	
Military Care	17 (47.2)
Overseas	4 (11.1)
Home Birth (military care)	1 (2.8)
Civilian Care	12 (33.3)
Both Military and Civilian Care	5 (13.9)
Overseas	1 (2.8)
Indian Health Service (IHS) care	1 (2.8)

*Note*: Some columns may not add to 100% due to missing data.

**FIGURE 1:**
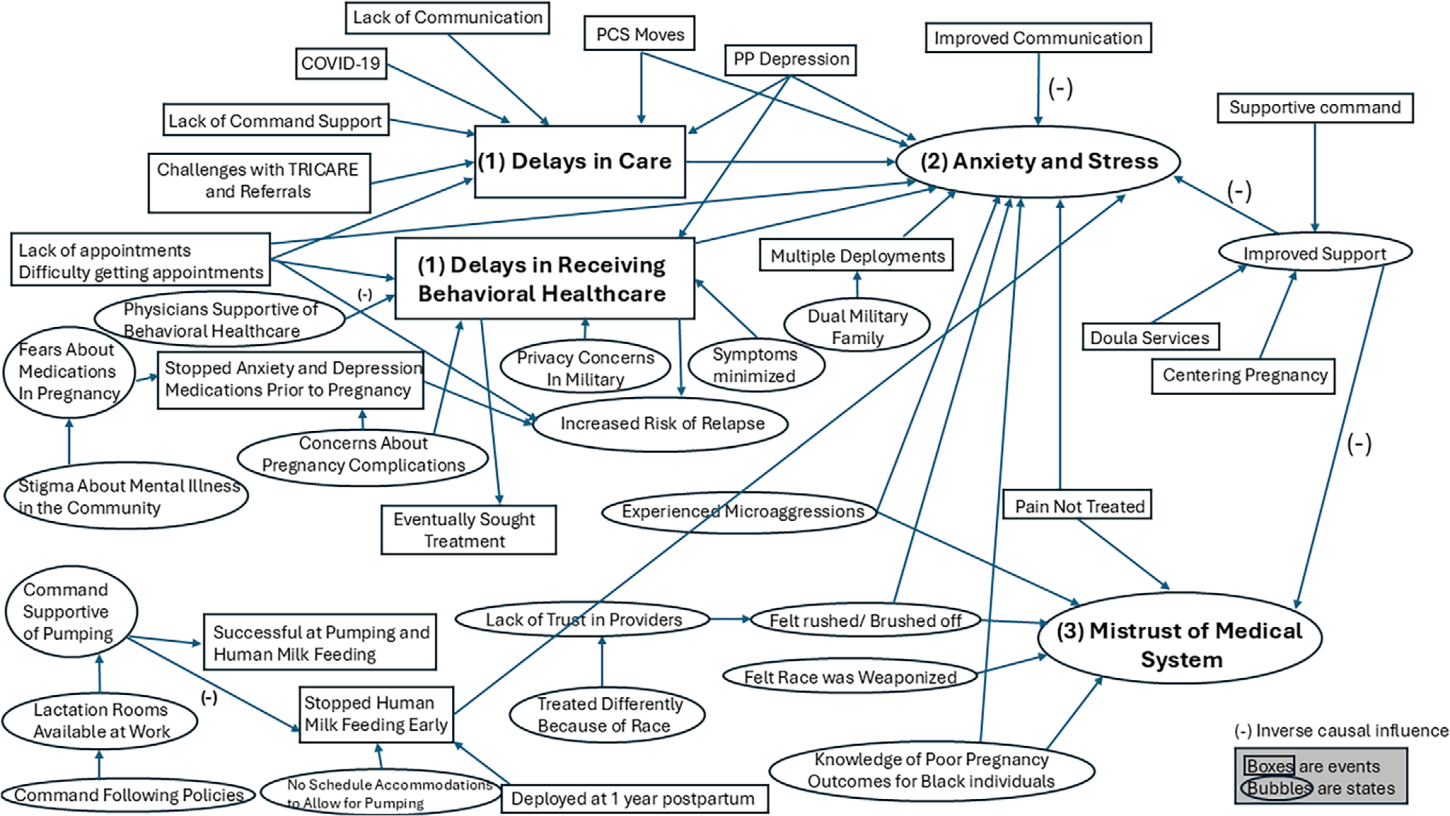
Causal network analysis.

**FIGURE 2:**
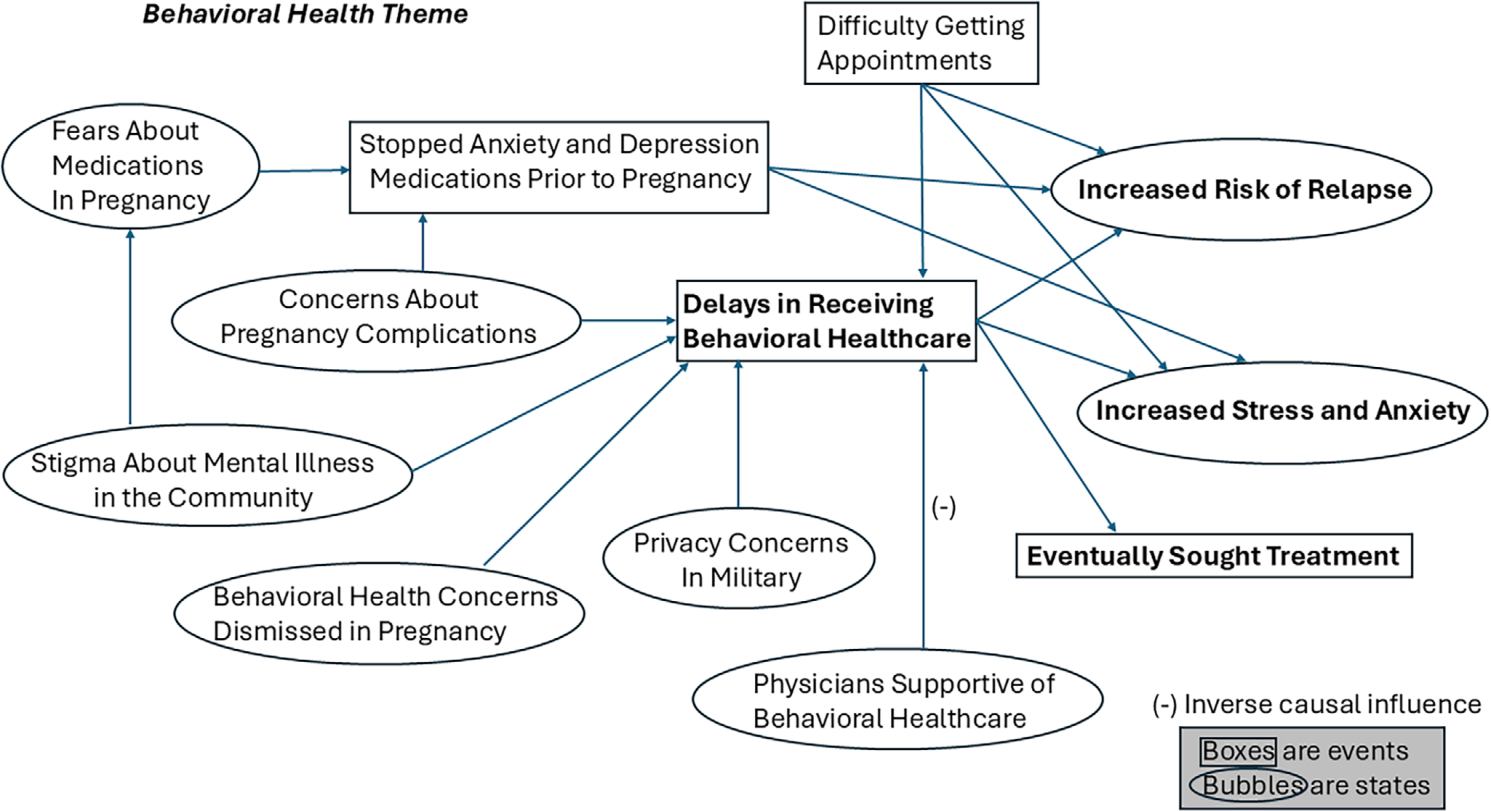
Behavioral health theme—causal network analysis.

**FIGURE 3:**
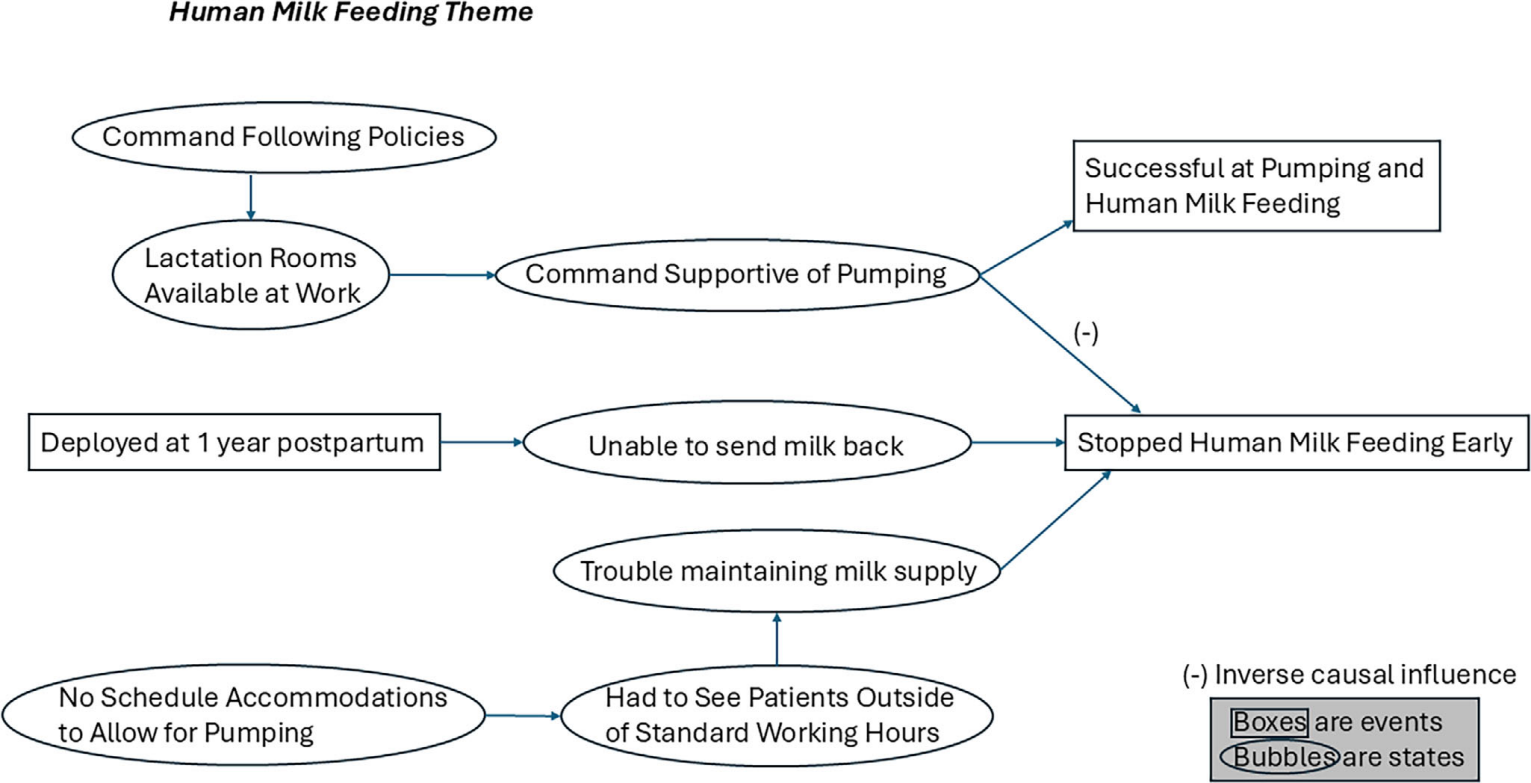
Human milk feeding theme—causal network analysis.

The resultant causal network is shown in Figure 1. The network can be organized into three main themes: (1) Delays in care (including delays in behavioral healthcare), (2) anxiety and stress, and (3) mistrust of the medical system. Additional causal networks centered around a behavioral health theme, which included (1) delays in care, (2) anxiety and stress, and (3) increased risk of relapse, as shown in Figure 2. Finally, a human milk feeding theme emerged with causal factors leading to success at human milk feeding and stopping early, as shown in Figure 3. Additional participant quotes are shown in Tables 2, 3, 4, 5.

**TABLE 2:** Existing concerns related to delays in care and potential solutions.

Delays in care	
Existing concerns potential solutions	
**Lack of appointments** “It was when I got here in a more fleet concentrated area [location with high amounts of military personnel and resources], like it was hard to get an appointment.” “I wasn't able to get an appointment, because they said they just didn't have any, and they never referred me out.” (Black, enlisted) “And so, even though I was active duty, pregnant in the second half of my pregnancy I had trouble getting appointments. After the first couple few routine appointments I figured out what you had to do was not call but rather I would walk down to their clinic sometimes a couple times a week to try to make my appointments and get an available appointment that aligned with when I was supposed to be seen.” (White, officer)	Improve staffing in Military Treatment Facilities (MTFs) Appropriate disengagements for purchased (civilian) care
**Challenges accessing care in civilian setting, including challenges with referrals** “So he's like my husband's beneficiary, but somehow the billing got entered as if he was my beneficiary. So, I like just until recently, I kept getting a bill for like almost a thousand dollars. I was like this doesn't make any sense. He has Tricare, like, this should be covered and he's like and everyone kept telling me he was ineligible for care.” (Indian, officer) “The referral process was atrocious. In order for the referral to go through, it had to go to a primary care manager (PCM) who had never seen me before, never met me. And in fact, any time I tried to call the PCM like the Occupational Health office, they were like, We're not allowed to talk to you, you can only talk to your OB and they would just route me back to the OB.” (Black, officer)	Patient navigators to help navigate the system Systemic improvements to referral system to make it easier for patients to transfer between direct and purchased care
**Lack of communication** “And I'm just thinking all kinds of what ifs and it was like, gosh, nobody from NICU has even come to help me and like, to introduce themselves and to tell me about the processes here, like, nothing and so I had to ask for that.” (Multi‐racial, Mexican/Indigenous, officer) “I was hoping for more education. Like, this is pregnancy, this is motherhood, this is stress, this is normal, this is not normal, and you should call me, and I really didn't get any of that.” (multiracial, Black, White, officer)	Improve communication between clinicians/care team and patients Patient portal communication Improve call center support
**Lack of command support** Command did not allow the patient to have a rental car when temporarily assigned overseas: “And so one of the reasons I feel like I had so many bleeds during my previa was because I was literally walking my ass from my [temporary lodging] to the hospital, almost a two mile walk each time I needed to get to triage.” (multiracial, Mexican/Indigenous, officer)	Improve support and temporary lodging for service members Educate commands and improve communication with healthcare clinicians
**Military ‘PCS, permanent change of station’ moves leading to gaps in care** “So then we PCS'd [permanent change of station (military move)] and so I went quite a while without care, which is ironic because the same thing happened to me with my oldest because we PCS'd in the middle of that pregnancy as well.” (White, officer)	Improve support for medical transitions, improve transitions between Tricare regions Improve appointment scheduling
**COVID‐19 pandemic leading to gaps in care** “I basically had a two‐month gap in a prenatal care. COVID was very prevalent at the time. Then I PCS'd [permanent change of station (military move)] to Japan. I couldn't get established with care until I was 19 weeks pregnant. So I had NO prenatal checks from 8 till 19 weeks. So I basically missed my entire first trimester.” (White, officer)	Emergency preparedness and policies for potential future pandemics to maintain continuity of care
**Privacy concerns and stigma with seeking help** “I wasn't ready to like, take a, the military blood test, because I was thinking, What if I miscarry? And now, I tell everybody that I miscarried, so there is just all these, like, concerns in that regard that you just wouldn't have on the civilian side.” (Black, officer) “She [her sister] also dealt with postpartum depression. but again, my culture is very, is not open about. mental health. It's a very taboo subject.” (Mexican, enlisted) “But the problem I ended up having with the whole situation. Is that because the clinic was so small, everybody in that clinic knew when I went on base to do my official pregnancy test, everybody then knew because of all the people I had to flipping talk to.” “So, it felt like a huge invasion of privacy, and to be honest, I really did delay going to get my blood tested on base for probably a week or two because I didn't want everybody to know.” (multiracial, Mexican, Indigenous, officer)	Policies supporting servicemember privacy Educational campaigns about the importance of care, including behavioral healthcare

*Note*: (‐) indicates a negative impact (decrease) on the variable.

Abbreviation: PCS,  Permanent Change of Station (military move to new duty station).

**TABLE 3:** Existing concerns related to anxiety, stress, and mistrust of the medical system and potential solutions.

Anxiety and stress	
Existing concerns potential solutions	
**Deployments increasing stress for families** “During my first pregnancy, [my partner] had deployed once, and then a few months after that we had to PCS [permanent change of station (military move)], so I was PCSing during my third trimester. That was hard, not only for me, but for him, moving everything.” (Mexican, enlisted)	Improve support for deploying families Improve doula support
**Knowledge of poor pregnancy outcomes in Black individuals** “I being Black, I have read so many horror stories and just reading statistics In general, I was tired of people telling me. I was going to a die in childbirth or be that I was gonna have subpar care simply because I'm Black.” (Black, officer)	Improve care and report outcomes stratified by race Education on efforts to decrease inequities The Centers for Disease Control “Hear her” Campaign available at: https://www.cdc.gov/hearher/index.html
**Differential care: microaggressions and minimization of concerns** “No one really took my pain seriously, and I don't know if it's because of that [race], I hope not.” (Mexican, enlisted) “So, I told you about the nurse who wouldn't check me when I told her, I was in pain and when she dismissed by saying so, when I have first mentioned ‘Hey, I'm supposed to be numb, right?’ Yeah. And I still feel, like, a lot of pain. She just discounted when I said I was in pain.” (Black, enlisted)	Improve clinician training and education to provide more sensitive and culturally humble care
**(‐) Improved communication** “The most positive part of my experience was the doctors just listening to me and really like not pushing me to do what I didn't want to do. They were good about listening. (white, spouse)”	Support improved communication between patients and clinicians
**(‐) Supportive commands** “ And on top of that, I also had my neuropsychiatrist, who worked within our active duty clinics, so he was always checking up on me, he always made time to see me. He was only there maybe once or twice a week. So I had like four different people helping me out and that alone made a big difference.” (Mexican, enlisted)	Encourage support through commands Educate commands on support Policies to provide servicemember support

Mistrust of Medical System	
Existing Concerns Potential Solutions	
**Treated differently due to race** “And maybe I might have like a hidden distrust, right? Of the medical system, because it just really hasn't been all that supportive.” (Mexican, officer)	Improve clinician training and education to provide more sensitive and culturally humble care
**Knowledge of poor pregnancy outcomes in Black individuals** “I've read up on the dehumanization of black women and medicine and things like that. I'm not uneducated when it comes to that, and I didn't want to assume that that was the case. But it was difficult for me to find another reason on two separate occasions. She just discounted when I said I was in pain.” (Black, enlisted) “With my daughter, I knew my race was a factor because I was in Georgia”. They have a horrible maternal mortality rate, all of the maternal deaths were women of color. So I, I was approaching that with caution and, um, I would say laced with some fear. (multiracial, Filipina/Black, officer)	Improve care and report outcomes stratified by race
**Differential care: microaggressions and minimization of concerns** “So she's saying something then something is wrong, but anytime I needed something, I had to go through him. And that was really, really frustrating because I'm speaking and I'm living the experience. Why are you not listening to me?” (Black, enlisted)	Improve clinician training and education to provide more sensitive and culturally humble care
**(‐) Support programs such as Doulas and Centering Pregnancy** “We did the centering program and I really enjoyed going to it because of the help. Because I was a first time Mom and also because I got to work with these ladies, right? And kind of grow up with these ladies. The military as an active‐duty person, is rather lonely from a female standpoint.” (Mexican, officer)	Continued and improved support for Centering Pregnancy and Doulas
**(‐) Support programs such as Doulas and Centering Pregnancy** “ I hired a doula because I wanted to kind of have somebody be able to back up my voice and help me express my needs, desires.” (white, spouse) “I had a postpartum doula…I couldn't imagine doing that without her those for eight weeks learning how to be a mom. You know, and just having her here, especially since I'm not near any family.” (White, enlisted)	Increase doula programs to support birthing individuals

*Note*: (‐) indicates a negative impact (decrease) on the variable.

Abbreviation: PCS, Permanent Change of Station (military move to new duty station).

**TABLE 4:** Existing concerns related to behavioral health and potential solutions.

Behavioral health	
Existing concerns potential solutions	
**Delays in receiving behavioral healthcare** “At the time me and my child's father was not talking and I was in Europe by myself so I had requested to come to a state that was close to my family so I can have the support for my pregnancy and they denied me for a while. And then it was like, I got last minute orders to come but I had to actually go see a therapist and talk to them and it was just a long process and they said, because of my rank, I couldn't leave so they wanted me to stay here with no support with having a baby. Yeah, that was that was a really big challenge on me.” (multiracial, Black, white, Puerto Rican, enlisted)	Increase access to behavioral healthcare Telehealth care to improve access
**Behavioral health concerns dismissed in pregnancy** “My therapist actually was really helpful, he was trying, and he made it work. But it was just me, because everybody else at my new command was just like you need to get over it, you chose to keep the baby.” (multiracial, Black, white, Puerto Rican, enlisted) “I'm over a year out [from pregnancy] and I've just kind of I think the past like four or five months is the first time that I've been kind of more stable from a mental health standpoint. They can't get you in more than once every four to six weeks for a therapy session.” (White, officer)	Improve clinician sensitivity to behavioral health needs Increase access to behavioral healthcare
**Concerns about medications, stopped early, afraid to take medications** “I think I was just mainly concerned that my baby would come out with any type of health issues. Was the medication harming my baby? I just didn't fully understand.” (Mexican, enlisted)	Improve education on safety of behavioral health medications in pregnancy, importance of treatment and risks of relapse
**Privacy concerns** “I did call Military OneSource and I like did the intake and then they never contact me again to get a counselor. But it was easy to get with in with the BHP [Behavioral Health Program] I was hesitant to go see them because I worked there and I didn't want people like, see me going into the Behavioral Health Office.” (White, officer)	Ensure servicemembers are aware of privacy protections for behavioral healthcare, including the Brandon Act, which allows for self‐initiated referrals for mental health evaluations to decrease stigma and access confidential care.
**(‐) Supported getting care** “I was receiving counseling at the time for ADHD and post‐traumatic stress from being in the military during a nuclear accident. So I'd already had counseling established with her. I was breastfeeding this whole time, and then at six months, I got really severe postpartum depression where it was impacting my activities of daily living. I was hesitant to start medications because I was breastfeeding and I wanted to continue to breastfeed, but my counselor was insistent on that I needed to initiate an agent because like the counseling was not doing it.” (multiracial, Filipina/Black, officer) Schedule accommodations so participant could get care for postpartum depression: “They were very understanding here. They let me go into the office for a few hours and then let me go home at like noon. So, they're very understanding and I'm very grateful for this command.” (multiracial, Black, White, Puerto Rican, enlisted)	Increase behavioral health support for patients postpartum including education on medication safety. Provide adequate support for accessing behavioral healthcare.

*Note*: (‐) indicates a negative impact (decrease) on the variable.

Abbreviation: PCS, Permanent Change of Station (military move to new duty station).

**TABLE 5:** Existing concerns related to human milk feeding and potential solutions.

Stopped human milk feeding early	
Existing concerns potential solutions	
**Deployed at 1 year postpartum** “I deployed for the first time when my oldest daughter was a year old.” (Indigenous/Lakota, enlisted) “I had to stop breastfeeding because I went to tech school training. And I think I had to stop at 11 months old. I had to stop because of that because they didn't have where you could pump, they didn't have the pumping locations mandatory yet and all that.” (Indigenous/Lakota, enlisted)	Improve and standardize service policies regarding deployment postpartum. Navy policy does not deploy until 12 weeks postpartum, while the Army postpartum deferment period is 4–12 months depending on the mission.
**No schedule accommodations for pumping as an active duty psychologist**: “They also said like, ‘We've also noticed that you're not keeping up with your patient care. You know, you're not meeting the requirements for a number of hours of patients.’ I'm like I'm confused, so you support breastfeeding in the workplace but there's not an opportunity to actually pump enough to keep my supply. And I have to maintain the same number of patient hours, which the only way to do that and pump would be for me to work outside of regular working hours. So, I ended up having to see patients before standard working hours and after standard working hours. So that I could keep up with the hours requirements for patient care which seemed absolutely ridiculous to me. Obvious, but they basically said like the breastfeeding instruction does not leave space for a diminished workload.” (White, officer)	Support for human milk feeding rooms and schedules to be flexible for pumping.
**(‐) Human milk feeding rooms available, command supportive** “We have a lactation room and I actually work right in front of the office. So, they'll let me go in there for like 20 minutes, every other hour and they knew I was in there and they just let me, you know, do my thing in there. They're very understanding when it came to that. Yes, I love it.” (multiracial, Black, White, Puerto Rican, enlisted)	Follow policies for human milk feeding rooms with command support to allow for pumping.

*Note*: (‐) indicates a negative impact (decrease) on the variable.

Abbreviation: PCS, Permanent Change of Station (military move to new duty station).

### 3.1 Delays in care (Table 3)

We identified key antecedent variables as systemic issues with TRICARE referrals, lack of available appointments including challenges accessing care in civilian settings, securing referrals, and finding a pediatrician for newborn care. Other contributing antecedent variables included a lack of communication, lack of command support, military permanent change of station (PCS) moves where patients had trouble re‐establishing care after their move, the COVID‐19 pandemic leading to gaps in care, and postpartum depression. They also experienced delays in receiving behavioral healthcare when their mood symptoms were minimized or “brushed off” by clinicians. Conversely, physicians who were supportive of behavioral healthcare were able to expedite care and improve patient experiences. Privacy concerns related to their military employment led some individuals to delay receiving behavioral healthcare. Furthermore, some individuals reported a stigma about receiving behavioral healthcare, which coupled with concerns about pregnancy complications led to them stopping depression and anxiety medications leading to an increased risk for relapses.

### 3.2 Anxiety and stress (Table 4)

Delays in care then resulted in increased anxiety and stress. Additional antecedent variables causing increased stress included dual military families experiencing multiple deployments, knowledge of poor pregnancy outcomes in Black individuals, and differences in care such as microaggressions (experienced by individuals who identified as Black, Indigenous, Mexican, and multiracial), feeling that their concerns were not taken seriously by clinicians, and inadequate pain treatment. Conversely, antecedent variables including improved communication between clinicians and patients, supportive commands and other programs that improved support (such as doula services and CenteringPregnancy classes) decreased anxiety and stress reported by participants.

### 3.3 Mistrust of medical system (Table 5)

Antecedent factors leading to mistrust of the medical system included being treated differently due to race, which led to a lack of trust in clinicians which was exacerbated when individuals felt their concerns were brushed off or not taken seriously. Black participants also reported feeling that their race was being weaponized, and the knowledge of poor pregnancy outcomes for Black individuals led to mistrust of the medical system. Individuals who experienced microaggressions and differential treatment of pain also had increased mistrust of the medical system, with these experiences reported by Black, Mexican, and Indigenous individuals. Improved support from doulas or CenteringPregnancy Programs led to improved trust in the medical system, and feelings of being supported with someone to advocate for them and give them advice during pregnancy and postpartum.

Related to the behavioral health causation model (Table 6) participants reported difficulty getting appointments, concerns about pregnancy complications, dismissal of behavioral health concerns, stigma and privacy concerns in the military led to delays in receiving behavioral healthcare, while supportive physicians decreased delays in receiving behavioral healthcare. Participants also reported stopping medications prior to pregnancy which led to an increased risk for relapses and increased stress and anxiety. Furthermore, delays and difficulty getting appointments also led to an increased risk of relapses and increased stress and anxiety, Figure 2.

Causal findings for the human milk feeding causation model (Table 7) identified that supportive commands with lactation rooms available at work were antecedent factors that enabled participants to be successful with human milk feeding, while deployments and lack of schedule and facility accommodations to allow for pumping were contributing factors to early cessation of human milk feeding. Challenges with human milk feeding were additional factors that increased anxiety and stress postpartum, Figure 3.

## 4 DISCUSSION

The unique experiences of birthing individuals in this study provide important insights into health and healthcare inequities in the MHS. Participants reported challenges related to care delays (Table 2), increased anxiety, stress, and mistrust (Table 3). Many themes and concerns centered around the ability of participants to access and utilize healthcare in the perinatal setting. Despite universal healthcare coverage, individuals report significant difficulties with referrals to the civilian network and availability of appointments in the MHS and civilian settings. The system to complete referrals was not easy to access and was especially challenging during military moves and when individuals needed care in both the direct care and purchased care markets. Improvements in the availability of appointments, ease of scheduling and referrals, and improved communication could decrease delays in care, decrease anxiety and stress, and decrease mistrust.

Current retrospective analyses of care and associated patient outcomes are limited by the fact that they only evaluate patients who sought and received care [16]. Individuals unable to schedule or keep appointments were not included and may have different experiences or outcomes. Thus, learning about individual experiences seeking and experiencing care provides an important perspective. Lack of appointment availability was commonly reported by participants, and improving MHS staffing shortages [17] remains a critically important step in improving access to care. Furthermore, half of active duty military installations are located within federally designated health professional shortage areas (HPSA) [18], and 35% of US counties are considered maternity care deserts [19], making it even more important to maintain military healthcare staffing in the setting of relative civilian staffing shortages. Recent reports highlight some of the ongoing staffing challenges, including data that 88% of Department of Defense facilities have nursing shortages [20].

Improving the systemic infrastructure to access appointments and increasing the availability of appointments (with improved staffing) in the MHS may also improve patient access to care. In a recent publication using the Joint Outpatient Experience Survey data from Army active duty servicemembers, the authors reported improvements in access to care with improved ease of scheduling appointments, the time between scheduling and actual visit, and wait time after scheduled appointment time [21]. The complexities of the referral and scheduling system in the MHS are also challenging for individuals scheduling perinatal and behavioral healthcare. Similar challenges have been reported in the Department of Veterans Affairs Health system, with comparisons to “finding your way through the labyrinth.” [22] Other opportunities to improve access to and completion of care include increasing patient navigators to assist patients with referrals and scheduling appointments and minimize inequities based on social factors such as age, education, and health. A recent study of access to care in the MHS using administrative data and patient satisfaction surveys reported that patient‐reported access to care was influenced by gender, age, education, and self‐reported health status, with increased age and good health status corresponding positively with improved access [23]. Potential solutions to improving access and satisfaction in the MHS are discussed in Tables 2, 3, 4, 5, and include improving call center performance for appointment scheduling and medical questions [24].

Challenges with scheduling appointments and completing referrals in the MHS are further exacerbated by frequent moves required of military servicemembers, and variable command support to attend medical appointments, which are unique social determinants of health for military service members and their families. Improving command support for patients and improving the ability to schedule appointments during moves would be beneficial. The recent COVID‐19 pandemic was another factor that contributed to delays in healthcare, and attention must be paid to maintaining care for routine services even in the setting of pandemics and other healthcare emergencies.

Participants reported that factors that decreased anxiety and stress included improved communication (Table 3), finding other individuals or peers who looked like them and faced similar struggles and supportive programs such as CenteringPregnancy group prenatal care and doula care. Increasing supportive care through support groups, doula access, and CenteringPregnancy are important components of improving care, and potentially mitigating racial inequities in the MHS. A recent randomized controlled trial of CenteringPregnancy group prenatal care found that increased participation in group prenatal care resulted in lower rates of preterm birth and low birthweight infants for Black individuals [25]. Similarly, use of doulas positively affects birthing outcomes including decreased need for analgesics, shorter labors, decreased cesarean delivery rates and improved patient satisfaction [26, 27]. Ultimately doula support may decrease health disparities and decrease costs for health systems [28], thus the MHS and Tricare could consider expanding current programs which offer six visits with a labor support visit. Increasing awareness of and use of support groups and doulas may further improve patient care for birthing individuals who are adapting to family and work changes following childbirth.

Participants also reported antecedent factors of microaggressions, differential treatment due to race, knowledge of poor outcomes for Black pregnant individuals and inadequate treatment of pain resulting in mistrust of the medical system. Such experiences then led individuals to question other aspects of care, or not to return for care with specific clinicians. Microaggressions stand out as an actionable item that healthcare workers can be aware of and address. Microaggressions are subtle, often unintentional, comments or actions that express bias or stereotypes, typically directed toward marginalized groups [29]. They may seem harmless, but can reinforce discrimination and make people feel excluded or devalued. These stigmatizing actions can create significant harm and mistrust in healthcare settings, and additional training and advocacy to decrease microaggressions in healthcare and society are important components of improving care. Addressing issues of systemic racism and microaggressions are important to improve trust and rebuild relationships in healthcare and mitigate health inequities. There is a biologic basis for the negative impacts of racism and microaggressions on health outcomes. The concept of “weathering” was developed by Dr. Arline Geronimus in the early 1990s [30]. She noted that societal racism can impact Black women resulting in “premature biological aging” ultimately leading to poor maternal health and pregnancy outcomes [30]. Allostatic load, or chronic stress, has further been shown to adversely contribute to the health of Black people [31]. Structural determinants of health (including structural racism, and aversive institutional policies) and social determinants of health such as food stability, education, environmental factors, and income, interact to influence Black maternal and neonatal outcomes [32]. Being Black itself isn't the issue. Black women exist at the intersection of race, gender and class, and this intersectionality of minoritized identities may also uniquely impact their well‐being [32, 33, 34].

Despite not asking direct questions about behavioral health, multiple behavioral health themes emerged from participant narratives with concerns such as privacy and the impact of medications and counseling on their pregnancy and their careers [35]. This is not surprising as perinatal mental health conditions affect an estimated 10%–20% of birthing individuals in the United States, with perinatal anxiety affecting another 8%–20% [36, 37]. Participants cited privacy concerns and some admitted to not being honest on screening forms, thus it is imperative that providers consistently complete screening and follow up negative responses with reassurances that patients may always return for help in the perinatal period if their mood changes. When participants had supportive interactions with clinicians, they found behavioral health services and medications to be helpful.

Participant experiences related to human milk feeding varied, with some individuals reporting command support, access to lactation rooms and adherence to military policies supporting human milk feeding and pumping. However, others reported challenges including deployments and lack of schedule and facility accommodations that resulted in early cessation of human milk feeding. Increasing adherence to policies and improved support of human milk feeding in the workplace are important components to ensure success and decrease stress and anxiety. While TRICARE covers outpatient lactation services, many military facilities have infrequent and unpredictable lack of inpatient lactation services which likely contributes to active duty women having lower breastfeeding initiation rates than the national averages [38]. This responsibility for lactation support falls onto already overburdened inpatient nursing staff, where positions are reportedly filled at 68% rates with OBGYN nursing amongst the lowest filled positions [39]. Further enhancement and availability of lactation support services and lactation consultants both inpatient and outpatient would also be beneficial.

Furthermore, this study included individuals who received care in military and civilian settings, and some cases where individuals received care in both settings. Positive and negative experiences existed across care locations and there was not a clear difference between care received in these settings or other participant characteristics. Individual experiences were categorized to develop the reported causal networks and answer the “why” question: Why do persistent healthcare inequities exist in the military health system? The answer includes multiple causes and outcomes, and based on our analysis, we have developed plausible causes of outcomes and probable outcomes from the individual causes [14].

The strengths of this study include the important findings and detailed experiences of individual participants. Multiple opportunities exist to improve access to care and timely appointments and to improve clinician interactions and communication with patients, and additional support for unique social determinants of health, including frequent military moves. Although military members have stable employment, they must balance work commitments and deployments, which may require additional support. The military health system covers 89,000 annual births, with approximately 75,000 births in the purchased care (civilian) market with Tricare insurance [5], and in this study, 47% of participants received military care, 33% received civilian care, and 13.9% received both military and civilian care, thus the challenges and experiences are relevant for both military and civilian obstetric clinicians. Another strength was the use of qualitative methodology, which includes investigators’ positionality, where personal backgrounds shape the research lens and findings. Throughout the study we reflected on our involvement as clinicians, military personnel, and mothers, and how this influenced the study. We engaged in critical self‐reflection and actively sought diverse perspectives from participants to increase our knowledge in this area. Finally, we report on the lived experiences of 36 participants, with an in‐depth and rich understanding of their individual experiences. These detailed experiences provide transferability [40] to other patients.

The limitations of this study include the possibility of different experiences in others and the possibility that other social determinants impacting participant health experiences were not discussed in our interviews. Another limitation was the use of multiple interviewers for this study, which was done to facilitate feasibility, and all interviewers received training and used an interview guide to standardize interviews. The interview guide (Supporting Information S2) was designed to assess overall participant birth experiences and did not specifically ask questions related to behavioral health, thus participant answers may have been different if specifically asked about behavioral health.

## 5 CONCLUSIONS

In summary, opportunities for improvements include addressing staffing shortages and availability of appointments, improved infrastructure and support for scheduling and referrals, improved communication, addressing systemic racism and microaggressions, encouraging command support, and maintaining the privacy of patients. Future research should investigate the effects of such improvements on patient experiences and outcomes in perinatal care.

## AUTHOR CONTRIBUTIONS

All authors conducted interviews that were included in this study. All authors contributed to manuscript drafting and editing. All authors read, edited, and approved the final manuscript.

## CONFLICT OF INTEREST STATEMENT

Some authors are federal employees or members of the armed forces. The views expressed are those of the authors and do not reflect the official policy or position of the U.S. Army Medical Department, Departments of the Army, Navy and Air Force, Defense Health Agency, Department of Defense, the Uniformed Services University of the Health Sciences, or the U.S. Government. The investigators have adhered to the policies for protection of human subjects as prescribed in 45 CFR 46.

Several of the authors are military service members. This work was prepared as part of their official duties. Title 17 U.S.C. 105 provides that ‘Copyright protection under this title is not available for any work of the United States Government.’ Title 17 U.S.C. 101 defines a United States Government work as a work prepared by a military service member or employee of the United States Government as part of that person's official duties.

## Supporting information



Supporting information

Supporting information

Supporting information

Supporting information

## Data Availability

Data from this project is not publicly available due to participant confidentiality
