# Supplementary material for: Perinatal healthcare inequities: A qualitative causal network analysis
Source: Pregnancy (Hoboken). 2025 May 4;1(3):e70021. doi: 10.1002/pmf2.70021 (PMC13344634; doi:10.1002/pmf2.70021)
Supplement: Supplementary file 1 [file PMF2-1-e70021-s001.docx]

Supplement 2. Interview Guide

Date:

Interview ID:

Interviewer:

**Introduction to the Interview**

First, my team and I would like to thank you for agreeing to participate in this study to explore the perinatal and birth experience of Tricare beneficiaries. The purpose of our study is to explore how you experienced your healthcare and to hear your story. We will use a series of questions to guide our conversation but feel free to tell me about your experiences in whatever way is comfortable for you.

The responses from all interviews will be combined for our report. You are free to refuse to answer any questions at any time in the interview and to stop the interview if you wish. If you have any questions about why I’m asking something, please stop me and ask. As a reminder, your responses will be recorded, transcribed and stored with a code number, which is designed to protect confidentiality. Any comments used in a report will not be identifiable. If you so desire, you will have 30 days from the interview to withdraw comments. In order to do so, you would need to contact Dr. Monica Lutgendorf, who is the only person who will have the key that connects your name with the number on the transcribed interview. If you choose to withdraw, at that time, the transcription will be permanently deleted from our collection.

Are you okay with this?

Please state your name:

Please confirm again that you are willingly participating in this interview and give permission for it to be recorded.

**Hit record on Google Meet (and transcribe), let participant know you are beginning recording**

***State your name (as interviewer), interview date and patient ID#**

I am going to start by getting to know you. Understanding your history and where you came from is important for us to have a complete picture. Tell me a little bit about your background and history?

Prompts:

- Where did you grow up?
- Who did you grow up with?
- How are you affiliated with the military?
- Tell me about your experience with healthcare before you joined the military?
- Tell me about your childhood.
- Did you have any challenges during your childhood?

As you know, we are interested in hearing about how birthing persons experience healthcare in the military surrounding their pregnancy and birth. We really want to hear about all the aspects of your care during your most recent pregnancy. Walk me through the story of your prenatal care, pregnancy and birth as you experienced it.

Prompts:

- What, if any, concerns did you have during your pregnancy, birth, and postpartum?
  - If concerns, how did you feel your concerns were addressed
  - If not concerns, what do you think helped you have no concerns?
- How did this pregnancy compare to other pregnancies/deliveries?
  - If first delivery can prompt for how did this compare to your expectations?
  - If not can probe for if other delivers where in military or not
- What were your expectations about where you would receive care and deliver?
  - How did your experience compare to your expectations
- Tell me about your postpartum care?

If you had to think about the most positive parts of your experience what comes to mind?

If you had to think about the most challenging part of your experience what comes to mind?

I would like to know a little bit more about you feel being in the military or having family in the military specifically impacted your experience. For example, some people have experienced a deployed spouse or PCS during pregnancy. What if any military related concerns or major life changes did you experience during your pregnancy?

Prompts:

- Any disruptions in your care?
- PCS moves?
- Changes in providers?
- Any difficulties scheduling appointments?
- Transfer to/from primary obstetric provider (transfer between military and civilian providers)

Thank you for sharing your pregnancy story. I know that it is not always easy to re-live some experiences. One thing that is reported in the literature is that some populations of woman have different outcomes from others. We want to explore some of the reasons why this might be happening. Tell me how you feel your race/ethnicity may or may not have played a role in the experience of the story you just told me.

Prompts:

- What specific examples can you think of where your race or ethnicity impacted care. This can be positive, negative, or neutral.
- What was that experience like?
- How did you feel in those moments?
- How did this impact you and your family?
- Are there other aspects of your identity that may have impacted your care?

The next part of this interview will focus a little bit more on your background and how that may have impacted how you experienced your pregnancy. Specifically, I would like to discuss more about your family experiences.

 What do you know of the experiences of your family members concerning their perinatal care and delivery experiences and what were your discussions with family members like regarding labor and delivery leading up to your delivery?

Prompts:

How did these decisions influence your experiences?

How did your background influence your experience?

I also wanted to learn more about your care post-birth. Please tell me how you experienced care following the birth of your baby?

Prompts:

- What specific examples can you think of where your race or ethnicity impacted care. This can be positive, negative, or neutral.
- What was that experience like?
- How did you feel in those moments?
- How did this impact you and your family?
- Are there other aspects of your identity that may have impacted your care?

Tell me more about the care of your newborn/infant.

Prompts:

- What specific examples can you think of where your race or ethnicity impacted care. This can be positive, negative, or neutral.
- What was that experience like?
- How did you feel in those moments?
- How did this impact you and your family?
- Are there other aspects of your identity that may have impacted your care?

I want to thank you for taking the time to share your story with me. Is there anything that I didn’t ask you that you feel would be helpful for me to know?

Additional questions/Demographics:

- Travel time to primary obstetric provider

- Travel time to delivery facility

- Active duty status?

- Prior service? When?

- What is your age____ Military rank ____/ Branch of service____ / Number of years service ____

- What is your partner’s military rank____/ Branch of service ____/ Number of years service ____

- How do you identify racially/ethnically?

- What languages do you speak?

**STOP recording and transcription and let participant know you have stopped recording and transcription**

**Thank you for taking the time to speak with me and share your story. Sometimes sharing your story can bring up strong emotions or feelings. This is a normal reaction. If you are feeling very sad or upset, or having lots of strong emotions from our interview, I can help you seek additional support or counseling services.**

How are you feeling?

Would you be interested in additional support or counseling?

Are you having thoughts of hurting yourself or anyone else? (yes answer would need a warm-handoff for emergency care and report to IRB)

****RESOURCES****

MILITARY ONE SOURCE	**800-342-9647**	<https://www.militaryonesource.mil/confidential-help/>

**Health and Human Services Maternal Mental Health Hotline**

The hotline is accessible by phone or text at 1-833-9-HELP4MOMS (1-833-943-5746) in English and Spanish. TTY Users can use a preferred relay service or dial 711 and then 1-833-943-5746.

The Maternal Mental Health Hotline is not intended as an emergency response line and individuals in behavioral health crisis should continue to contact the National Suicide Prevention Lifeline at 1-800-273-TALK (8255).

**Interviewer Reflection on the Process/Interview Overall** **Please note any thoughts below:**
