# Supplementary material for: Perinatal healthcare inequities: A qualitative causal network analysis
Source: Pregnancy (Hoboken). 2025 May 4;1(3):e70021. doi: 10.1002/pmf2.70021 (PMC13344634; doi:10.1002/pmf2.70021)
Supplement: Supplementary file 2 [file PMF2-1-e70021-s003.docx]

Supplement 1 – Study Team Reflexivity Statements

EB is an active duty pediatric hematologist whose pregnancy experiences shaped her lens, CMD is an active duty neonatologist and qualitative researcher whose own experience of pregnancy within the MHS helps to inform her understanding and interpretation of the participant experience, CMD is an active duty neonatologist and qualitative researcher whose own experience of pregnancy within the MHS helps inform her understanding and interpretation of the participant experience, GH who is a white, Jewish, neonatologist whose background and experience help shape her desire to discover and mitigate causes of healthcare disparities, SMF is an active duty obstetrics and gynecology resident physician who is Black and a healthcare inequities researcher, AK is a qualitative researcher whose experiences as a mother and researcher shaped her lens, MAL, who is an active duty maternal-fetal medicine physician who is multiracial and interested in understanding the patient experience and decreasing healthcare inequities, MGM, who is an active duty gynecologic surgery and obstetrics resident physician who is a white and interested in improving patient’s experience of prenatal and postpartum care, TCP is an active duty, Black cisgender female reproductive endocrinology and infertility physician whose personal birth experiences and research in health inequities may impact her interpretation of participants’ stories, TRR is an active duty, Black certified nurse midwife, whose personal birth experiences and background in maternal-child health influenced her connections and understanding of participant birth stories, CNS is a military nurse veteran whose personal experience of childbirth and of providing healthcare to military families influences the way in which she interpreted this information, RJV is an active duty military physician who identifies as a Black cisgender woman, experiences within pediatrics, neonatal-perinatal medicine and as a researcher in health disparities may shape the lens through which she viewed the participants’ stories, BWN is an active duty general medical officer who identifies as Black, whose lens was shaped by experiences in multiple military medical facilities and as an operational medicine provider.
