# Supplementary material for: Perinatal healthcare inequities: A qualitative causal network analysis
Source: Pregnancy (Hoboken). 2025 May 4;1(3):e70021. doi: 10.1002/pmf2.70021 (PMC13344634; doi:10.1002/pmf2.70021)
Supplement: Supplementary file 3 [file PMF2-1-e70021-s004.docx]

Supplement 3. Steps in Causal Network Analysis^13^

| **Code Example (→ means “leads to”)** | | |
| --- | --- | --- |
| **Interview quotes** | **Codes** |  |
| “When I got it replaced that was the first time that I had a terrible medical experience because I replaced got the same brand, it was Mirena. but for some reason, They say it was the same one, but I was bleeding for days like heavy bleeding after they placed the IUD.”  “So, first and foremost scheduling was hellacious, right. I know y'all just put this in, something is wrong. can you take it out, maybe put a different one in. I got told that my comfort is not a reason is not priority, and that I would have to wait and that the soonest that they could see me would be their basically their third visit out because they were already booked in that.”  “And the one it really made me sad because it was a black woman doctor I was super mad and she was like, you're making a big deal out of something people bleed all the time and I was like, yes, but I know my body and she was like, “Do you know your body because the last time you got an IUD, you were 24 years old and now you're 28 or 29, how old I was. And she was like your body changes, maybe this is your new body's reaction and it was so dismissive.” | ^1^Requested Mirena removal due to bleeding → terrible experience, physician acted like she didn’t know her body since it was several years from her last IUD placement → felt dismissed  ^2^Challenges scheduling → told her comfort was not a priority, and she would have to wait until the next available appointment |  |
| **Analysis (attribution sequence)** | | |
| **Antecedent conditions →** | **Mediating Variables →** | **Outcome Category** |
| Requested IUD removal  Concerns not taken seriously  Challenges scheduling – Lack of appointments | Dismissive - Acted like she didn’t know what she was talking about  Her comfort was not a priority  Delays in care | Sad, Angry  Anxiety  Mistrust |
| **Causation Model** | | |
|  | | |
